# Two new species of the ground beetle subgenus *Sadonebria* Ledoux & Roux, 2005 (Coleoptera, Carabidae, *Nebria*) from Japan and first description of larvae of the subgenus

**DOI:** 10.3897/zookeys.578.7424

**Published:** 2016-04-07

**Authors:** Kôji Sasakawa

**Affiliations:** 1 Laboratory of Zoology, Department of Science Education, Faculty of Education, Chiba University, 1-33 Yayoi-cho, Inage-ku, Chiba 263-8522, Japan

**Keywords:** Cryptic species, endophallus, geometric morphometrics, larval morphology, male genitalia, Nebria quinquelobata sp. n., Nebria sadona, Nebria yatsugatakensis sp. n., taxonomy

## Abstract

*Sadonebria* Ledoux & Roux, 2005 is one of the more diverse subgenera of the genus *Nebria* Latreille, 1802 in East Asia, and its taxonomy remains unrevised at the subgeneric and specific levels. In this paper, two new species of this subgenus are described from Japan. *Nebria quinquelobata* **sp. n.** is described from Mt. Myôkô and is externally similar to *Nebria saeviens* Bates, 1883, to which specimens of this new species previously had been assigned. *Nebria yatsugatakensis* **sp. n.** is described from the Yatsugatake Mountains and is externally similar to locally adjacent species that had been recognized as *Nebria sadona* Bates, 1883 and were recently revealed as separate species. Both new species are distinguished by morphological (the shape of the endophallus) and morphometric (geometric morphometrics of the pronotum and aedeagus) features. For *Nebria yatsugatakensis*, the morphology of all larval instars is described based on specimens reared from eggs laid by collected adults. These results, together with previous studies of the species-level taxonomy of *Sadonebria* and larval morphology of other *Nebria* subgenera, suggest (i) the utility of geometric morphometrics in species-level taxonomy; (ii) the importance of larval secondary setae in the subgeneric taxonomy of the genus *Nebria*; and (iii) the presence of further cryptic species in *Sadonebria*.

## Introduction

**Figure 1.**
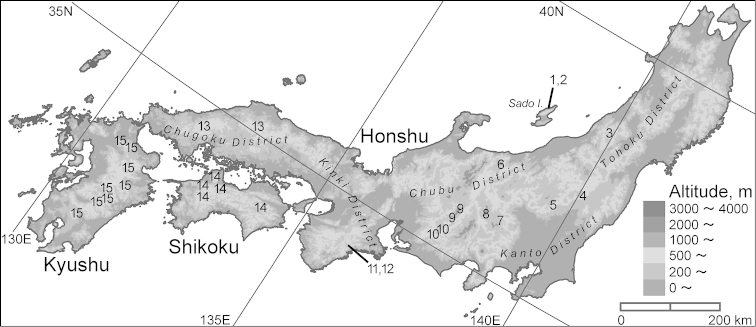
The distribution of Nebria (Sadonebria) spp. in the Japanese Archipelago; *Nebria chinensis* Bates, which is distantly related to the remaining species, and samples that are not identified by genital morphology are not included [modified from Sasakawa and Toki (2011)]. **1** *Nebria sadona sadona* Bates **2** *Nebria saeviens* Bates **3** *Nebria asahina* Sasakawa **4** *Nebria nasuensis* Sasakawa **5** *Nebria sadona leechi* Bates **6** *Nebria quinquelobata* sp. n. **7** *Nebria chichibuensis* Sasakawa **8** *Nebria yatsugatakensis* sp. n. **9** *Nebria kiso* Sasakawa **10** *Nebria mikawa* Sasakawa **11** *Nebria sadona ohdaiensis* Nakane **12** *Nebria tenuicaulis* Sasakawa & Kubota **13** *Nebria jakuchisana* Sasakawa **14** *Nebria shikokuensis* Sasakawa; 15: *Nebria trifida* Sasakawa.

*Sadonebria* Ledoux & Roux, 2005 is an endemic East Asian subgenus of the genus *Nebria* Latreille, 1802 (Coleoptera, Carabidae). To date, 15 species-group taxa (13 species and two subspecies) have been described in this subgenus. Among the 15 taxa, *Nebria chinensis* Bates, 1872, which has developed hind wings, is widely distributed in China, Korea, and Japan (Farkač and Janata 2003). The remaining taxa, which have atrophied hind wings and are flightless, are endemic to Taiwan (*Nebria niitakana* Kano, 1930) and Japan (the remaining 13 taxa). Of the 13 Japanese taxa, nine were previously considered *Nebria sadona* Bates, 1883 due to marked similarities in external morphology but were recently separated based on the shape of the endophallus (a membranous inner sac everted from the aedeagus of male genitalia) (Sasakawa and Kubota 2006; Sasakawa 2008, 2009, 2010; Sasakawa and Toki 2011), a character that had been insufficiently examined by previous authors (e.g., Habu 1962; Uéno 1985). According to the latest comprehensive study by Sasakawa and Toki (2011), a major diversification of *Sadonebria* in the Japanese Archipelago occurred in the area east of Kinki, Honshu, where the species diversity of this group is highest (Fig. 1). However, taxonomic studies of *Sadonebria* in this area are still insufficient, and there are many localities from which no specimens have been examined. Moreover, the recent studies were predominantly based on comparative morphology of the single male genital character, and other morphological and genetic characters remain unexamined. It is important to address these issues to better understand the diversification of *Sadonebria* in the Japanese Archipelago.

In this paper, two new species of *Sadonebria* are described from the area east of Kinki, Honshu, Japan. As with the recently described species, the new species are separated from known species based on the shape of the endophallus. Here, in addition to comparative morphology of this genital character, geometric morphometrics of external and genital characters are also performed. This morphometric technique can effectively evaluate subtle morphological differences, which are difficult to detect by traditional morphometrics, and is now widely used for the analysis of morphometric data (e.g., Sha et al. 2016; Sasakawa 2016; Kosuda et al. 2016). Therefore, it may be useful for species-level taxonomy of *Sadonebria*, members of which have markedly similar external and genital (other than the endophallus) morphologies. For one of the new species, the morphology of all larval instars is also described based on specimens reared from eggs laid by collected adults; this is the first report of larval morphology for the subgenus *Sadonebria*. The implications of these results for the taxonomy of both the subgenus *Sadonebria* and the genus *Nebria* are discussed.

## Materials and methods

### Morphological comparison and description

Information regarding comparisons of adults of related species was obtained from Sasakawa (2008, 2009), Sasakawa and Kubota (2006), and Sasakawa and Toki (2011), which describe key characters of the species, such as the male endophallus, based on their type materials (species other than *Nebria saeviens*) or materials from the type locality (*Nebria saeviens*). For *Nebria saeviens*, the following additional materials from the type locality (Sado Island) were also examined: 5♂6♀, the upper reaches of the Kuchi river, Sado-shi, Niigata Prefecture, 10.vi.2010, Naoyuki Shibata leg. These specimens were also used in subsequent geometric morphometric analyses. Terminology of the male endophallus followed Sasakawa (2009).

To obtain larval specimens, adults of *Nebria yatsugatakensis* sp. n. were reared in plastic boxes (17.0 × 8.5 × 4.5 cm) following the technique described in Sasakawa (2011). The adults were collected at the type locality on 4–5 September 2010. Eggs were laid in the mud of rearing boxes from 5–15 September and were left in place until hatching. To simulate cooling autumn temperatures, the adults and eggs were reared under gradually decreasing temperatures: 18°C (5 September–10 October), 15°C (11–22 October), 10°C (23–26 October), and 5°C (27 October–3 November). The photoperiod was maintained at 8:16 h light:dark, and *Tenebrio molitor* larvae (cut into pieces) were provided as a food source. The eggs hatched between 30 October and 3 November and then were moved to Petri dishes (3.5 cm diameter, 1.0 cm high) filled with 0.5 cm moistened garden soil, after which they were reared at a constant temperature of 5°C or 10°C and checked daily for development. In total, 15 first-instar, 16 second-instar, and 14 third-instar specimens of *Nebria yatsugatakensis* sp. n. were obtained. For detailed morphological observations and measurements, five first-instar, four second-instar, and five third-instar specimens were dissected and mounted on permanent microscope slides in Euparal medium, according to the methods described by Grebennikov and Maddision (2005). Other specimens were observed and preserved in 70% ethanol. The notation used for the setae and pores followed Bousquet and Goulet (1984) and Bousquet (1985).

The examined specimens were deposited in the collections of the National Institute for Agro-Environmental Sciences, Tsukuba, Japan (NIAES), the Laboratory of Forest Zoology, Graduate School of Agricultural and Life Sciences, University of Tokyo, Tokyo, Japan (FZUT), and those of the author (KS).

### Geometric morphometrics

**Figures 2–3.**
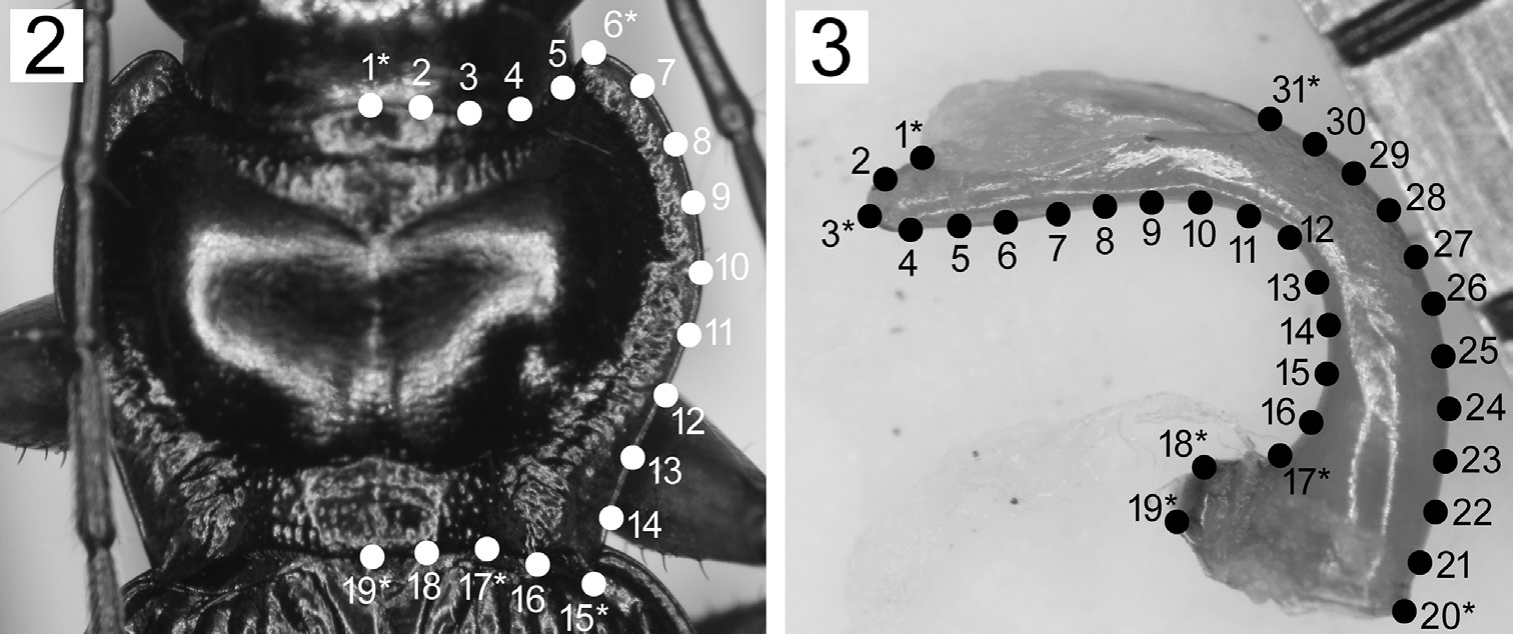
Positions of landmarks (numbers with asterisks) and semi-landmarks (numbers without asterisks) on (**2**) a pronotum in the dorsal view and (**3**) an aedeagus in the right lateral view. Landmarks indicated in (**2**) are as follows: (1*) anterior end along the median line; (6*) apex of anterior angle; (15*) apex of posterior angle; (17*) proximal end of posterior angle; and (19*) posterior end along the median line. Landmarks indicated in (3) are as follows: (1*) apical end of membranous portion; (3*) aedeagal apex; (17*) basal end of ventral side of cylindrical part; (18*) apex of the prominence between landmarks 17 and 19; (19*) basal end of ventral side; (20*) basal end of dorsal side; and (31*) basal end of membranous portion. Semi-landmarks were plotted along the curvature at regular intervals between landmarks.

Geometric morphometrics were performed for the dorsal view of the pronotum and the left lateral view of the aedeagus of male genitalia using the following materials: *Nebria quinquelobata* sp. n., 3♂1♀, *Nebria saeviens*, 5♂6♀; *Nebria yatsugatakensis* sp. n., 3♂6♀; and *Nebria chichibuensis* Sasakawa, 2010, 1♂2♀. The materials of species other than *Nebria saeviens* are type series of each species. Scaled digital images were obtained using a charge-coupled device camera attached to the microscope, after adjusting the tilt in the horizontal direction of materials. The pronotum was maintained with the anterior and posterior ends (landmarks 6 and 15 in Fig. 2 and corresponding points of the left lateral side) in the same horizontal plane, and the aedeagus was placed such that the ventral margin (including sub-landmarks 5–10 in Fig. 3) near the membranous portion was maintained horizontally.

For the pronotum, five landmarks and 14 semi-landmarks were identified (Fig. 2). For the aedeagus, seven landmarks and 24 semi-landmarks were identified (Fig. 3). The coordinates were digitized using the software tpsDig version 2.17 (Rohlf 2013a). Using the software tpsRelw version 1.53 (Rohlf 2013b), the raw coordinates were converted to Procrustes coordinates, in which variations due to rotation, position, and size were removed with semi-landmarks being “slid” along the contours. Relative warp analysis and visualization of shape differences also were performed using this software.

To statistically evaluate shape differences, Procrustes ANOVA with 10,000 permutations was performed using the function procD.lm in the R package geomorph (Adams and Otarola-Castillo 2013). In both the pronotum and aedeagus analyses, all relative warp scores were included as response variables. For the pronotum, species, sex, and their interaction were included as explanatory variables. For the aedeagus, *Nebria chichibuensis* was excluded from the analysis because of its small sample size (n=1), and species was included as the explanatory variable. If needed, post hoc pairwise comparisons were performed using the Bonferroni-corrected significance level.

## Results

### Taxonomy

The two new species described here were distinguished from known species based on the shape of the endophallus. This result was complemented by that of geometric morphometrics, which is described later.

The two new species shared the following adult morphological character states. Habitus slender. Hind wings atrophied. Chaetotaxy as in other consubgeneric species (Sasakawa and Kubota 2006). Dorsal surface shiny and almost black; mouth appendages and antennae dark brown. Pronotum cordate and convex; lateral margins reflexed throughout; hind angle acute; laterobasal impression large and deep; median line impressed in the middle, reaching both the anterior and posterior margins; surface of central part almost smooth; surface of the anterior and posterior margins punctate; surface of the lateral margins sparsely punctate and/or shallowly, transversely wrinkled. Elytra oblong, widest behind the middle; four to seven dorsal pores on interval 3. Aedeagus slender and strongly arcuate, with simple apex. Endophallus stout, with four types of lobes on the surface, namely the laterobasal, lateroapical, dorsobasal, and dorsoapical lobes.

#### Nebria (Sadonebria) quinquelobata sp. n.

Taxon classificationAnimaliaColeopteraCarabidae

http://zoobank.org/AB3FF90A-C284-42D6-AFF9-1F228F14C999

Figs 4 , 8

Nebria saeviens (part): Uéno (1985): 56, fig. 11.
Nebria (Orientonebria) saeviens (part): Farkač and Janata (2003): 94.
Nebria (Sadonebria) saeviens (part): Ledoux and Roux (2005): 824, fig. 621; Yoshitake et al. (2011): 38.

##### Type materials.

Holotype: ♂ (NIAES), “X. 13, 1965 / Mt. Myoko / Niigata P. / K. BABA” [type locality: Mt. Myôkô, Myôkô-shi, Niigata Prefecture, Japan]. Paratypes: 2♂1♀ (NIAES), “Sasagamine [in Japanese] / S-Echigo / 28.VIII, 1966 / Col. K. Baba”.

##### Etymology.

The specific name derives from the Latin adjectives *quinque*- (five of) and *lobatus*, -*a*, -*um* (with lobes) and refers to the ventral view of the male endophallus (Fig. 8c).

**Figures 4–7.**
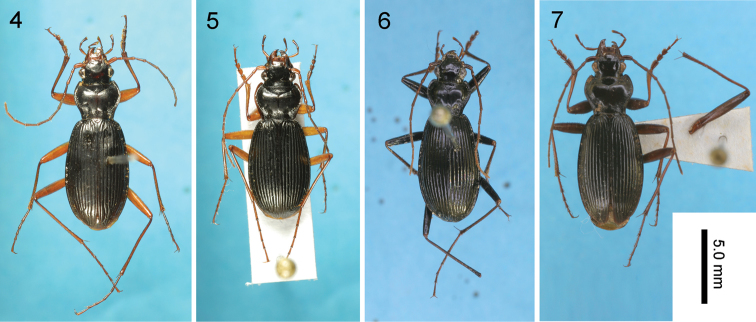
Dorsal view of Nebria (Sadonebria) spp. **4** *Nebria quinquelobata* sp. n., holotype male **5** *Nebria saeviens*, male from type locality **6** *Nebria yatsugatakensis* sp. n., holotype male **7** *Nebria chichibuensis*, holotype male.

**Figures 8–11.**
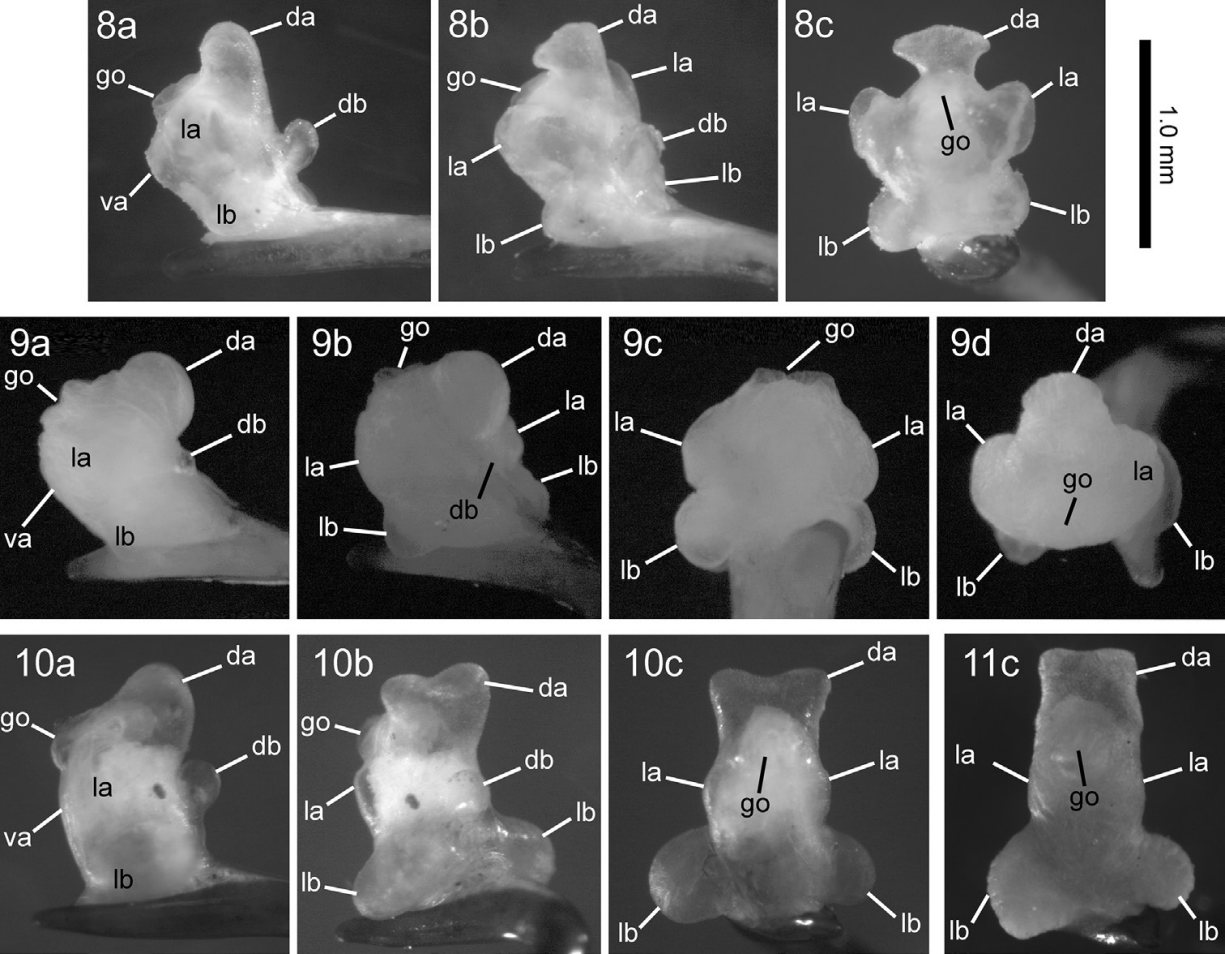
Left lateral view (**a**), left dorsolateral view (**b**), ventral view (**c**), and posterior view (**d**) of the endophallus of Nebria (Sadonebria) spp. **8** *Nebria quinquelobata* sp. n., holotype male **9** *Nebria saeviens*, male from type locality **10** *Nebria yatsugatakensis* sp. n., holotype male **11** *Nebria chichibuensis*, holotype male. go, gonopore; da, dorsoapical lobe; db, dorsobasal lobe; va, ventroapical lobe, la, lateroapical lobe; lb, laterobasal lobe.

##### Diagnosis.

Similar to *Nebria saeviens* (Fig. 5) in having yellowish brown legs but distinguished by the shape of the endophallus (Figs 8, 9).

##### Description.

*External structures*: Body length (including mandibles): ♂, 11.7–12.3 mm (mean ± SD: 12.0 ± 0.30 mm, n = 3); ♀, 13.1 mm (n = 1). Head with a reddish-brown patch between the eyes; pronotum reddish brown on the central part and dark brown at the lateral margins; head and other pronotal parts and elytra black; legs yellowish to light brown.

*Male genitalia*: Laterobasal lobes and lateroapical lobes both largely swollen and widely rounded, with the former slightly smaller than the latter; the dorsoapical lobe similar in size to the laterobasal lobe and bifurcated in a T shape at the apex; dorsobasal lobe distinct but smaller than the other lobes.

#### Nebria (Sadonebria) yatsugatakensis sp. n.

Taxon classificationAnimaliaColeopteraCarabidae

http://zoobank.org/FB855039-F899-4CE9-8B07-8EB61FB2B249

Figs 3 , 7 , 12–19 , 20–21 , 22–28 , 29–30

Nebria sadona (part): Uéno (1985): 56, fig. 10.
Nebria (Orientonebria) sadona (part): Farkač and Janata (2003): 94.
Nebria (Sadonebria) sadona (part): Ledoux and Roux (2005): 822, fig. 618; Yoshitake et al. (2011): 37.

##### Type materials.

Holotype: ♂ (FZUT), Tamagawa, Minoto, Chino-shi [the Yatsugatake Mountains], Nagano Prefecture, Japan (35°58'52.6"N, 138°18'29.4"E; ca. a.l.t. 1600 m), 4-5.ix.2010, K. Sasakawa leg. Paratypes (KS): 2♂6♀, same locality (2♀, 15-16.ix.2009, K. Sasakawa & H. Ikeda leg.; 2♂4♀, 4-5.ix.2010, K. Sasakawa leg). Larval specimens (KS) are not designated as type materials.

##### Etymology.

The specific name is derived from the Yatsugatake Mountains, the type locality of the new species.

##### Diagnosis of adult.

Similar to locally adjacent species, such as *Nebria chichibuensis* Sasakawa, 2010 (Figs 7, 11; see also Fig. 1), but distinguished by the shape of the endophallus (for example, the dorsoapical lobe is clearly Y-shaped in *Nebria yatsugatakensis* but not in *Nebria chichibuensis*). Distinguished from *Nebria sadona* by a pronotum line that reaches both the anterior and posterior margins, a feature that is absent in *Nebria sadona* near the anterior and posterior margins (Sasakawa 2008).

##### Description of adult.

*External structures*: Body length (including mandibles): ♂, 11.7–12.9 mm (mean ± SD: 12.2 ± 0.58 mm, n = 3); ♀, 12.8–13.9 mm (mean ± SD: 13.3 ± 0.41 mm, n = 6). Head without a reddish-brown patch between eyes; pronotum entirely black, but lateral margins dark brown in some specimens; legs dark brown, except for the femora, which are brownish black.

*Male genitalia*: Laterobasal lobes markedly swollen; laterapical lobes superficial; dorsoapical lobe large, similar in size to the laterobasal lobe, with the apex bifurcated in a Y shape; dorsobasal lobe distinct but smaller than the laterobasal and dorsoapical lobes.

##### Diagnosis of larvae.

In older instars, distinguished from congeneric species by numerous setae on the entire surface of thoracic nota and abdominal tergites (see Discussion).

##### Description of larvae.

*Characters present in all instars*: head capsule dark-brown to brownish black, with lighter ventral side; mouthpart appendages and legs brown to light-brown; urogomphi light- to dark-brown; other scletites on thorax and abdomen gray to brownish-gray; membranous parts grayish white. Most primary setae and pores present, but at least the following ones absent: FR_8_, FR_9_, LA_4_, PR_7_, ME_2_, TE_4_, TE_6_, TE_9_, EM_1_, FR_f_, PA_n_, CO_d_, TE_b_, and PY_e_. Head capsule oval, widest at stemmata. Frontale U-shaped at base, with posterior end at the level of basal 1/3 of head capsule; nasale prominent, with three pairs of large projections; adnasale sloping posterolaterally. Parietale with six stemmata; cervical groove absent; coronal suture present. Antennae longer than mandible; antenomeres I and III subequal in length, longer than II and IV. Mandible slender and arcuate, with sharp apex; terebra without tooth-like processes; retinaculum as long as the width of the mandible at the level of MN_1_ and curved inward, with sharp apex. Maxilla with stipe as long as palpomeres III and IV combined; palpomere II and III subequal in length, shorter than IV, and longer than I; membranous notch absent. Labium with cordate prementum and elongated ligula; palpomere I longer than ligula but shorter than palpomere II. Thoracic nota and abdominal tergites transverse; notal carina of meso- and metanotum and abdominal tergal carina distinct. All legs with two unequal claws, with the anterior claw longer than the posterior one. Urogomphi slender, longer than head capsule.

*Characters restricted to first-instar larvae*: head width 1.16–1.22 mm (mean ± SD: 1.20 ± 0.02 mm, n = 5). Urogomphi 1.59–1.72 mm (mean ± SD: 1.66 ± 0.05 mm, n = 5). Secondary setae present on maxilla (9–11 for gMX), pronotum (one on central part, and one on posterior part), and abdominal epipleurite (one on central part). Head capsule with longitudinal, keel-like egg-bursters. Antennomere II almost cylindrical, subequal in length to IV. Maxillary palpomere III less than half the length of IV. Pronotum with indistinct notal carina. Urogomphi fused to tergite IX.

*Characters restricted to older instars*: Head width 1.50–1.52 mm (mean ± SD: 1.51 ± 0.01 mm, n = 4) in second instar; 1.80–1.89 mm (mean ± SD: 1.85 ± 0.04 mm, n = 5) in third instar. Urogomphi 2.08–2.27 mm (mean ± SD: 2.19 ± 0.08 mm, n = 4) in second instar; 2.51–2.94 mm (mean ± SD: 2.77 ± 0.16 mm, n = 5) in third instar. Secondary setae present on antennomere I (two on inner side), maxilla (14–19 for gMX, and two near MX_b_), labium (one behind LA_3_), frontale (absent or less than two near FRe and/or about three around FR_1–3_), and epimeron (absent or less than two); parietale, thoracic nota, abdominal tergites, epusterna, epipleurites, pleurites, sterna, pygidium, urogomphi, legs except claws with numerous secondary setae on entire surface. Antennomere II longer than IV, with the distal end being distinctly wider than the proximal end. Maxillary palpomere III more than half the length of IV. Pronotum with notal carina distinct. Urogomphi not fused to tergite IX.

##### Remarks.

For larvae reared at 5°C, the number of days (mean ± SD) of the first and second instars were 25.50 ± 2.25 (n = 16) and 42.50 ± 1.87 (n = 6), respectively. For larvae reared at 10°C, the durations of first and second instars were 15.29 ± 0.61 (n = 14) and 22.13 ± 2.30 (n = 8).

### Geometric morphometrics

Relative warp analyses generated 27 and 11 scores for the pronotum and the aedeagus, respectively. To visually capture the results, scatter plots based on the first two scores were created (here referred to as RW1 and RW2). In the pronotum, RW1 accounted for 31.9% of the total variance and was mainly associated with the sinuation of the basal part of the lateral margin (landmarks 12–15). RW2 accounted for 20.0% of the total variance and was mainly associated with the sinuation of the lateral side of the posterior margin (landmarks 15–17) and relative size of the apical half. On the scatter plot, four species with overlapping areas were segregated (Fig. 31). Results of Procrustes ANOVA revealed significant effects of species (*F*_3,19_ = 20949, *p* < 0.001) and sex (*F*_1,19_ = 12805, *p* = 0.022) and no significant effects of the interaction term (*F*_3,19_ = 1.39, *p* = 1.000). Subsequent analysis with species as the only explanatory variable revealed that the overall difference was significant (*F*_3,23_ = 37.56, *p* < 0.001). Post hoc pairwise comparisons indicated that the differences between *Nebria quinquelobata* and *Nebria chichibuensis*, and between *Nebria yatsugatakensis* and *Nebria chichibuensis* were not significant (*F*_3,6_ = 0.808, Bonferroni-corrected *p* = 0.225; and *F*_3,8_ = 0.625, Bonferroni-corrected *p* = 0.256; respectively) but the other four between-species differences were significant (*Nebria saeviens* vs. *Nebria quinquelobata*: *F*_3,11_ = 0.871, Bonferroni-corrected *p* = 0.004; *Nebria saeviens* vs. *Nebria yatsugatakensis*: *F*_3,16_ = 1.710, Bonferroni-corrected *p* < 0.001; *Nebria saeviens* vs. *Nebria chichibuensis*: *F*_3,10_ = 1.761, Bonferroni-corrected *p* = 0.003; *Nebria quinquelobata* vs. *Nebria yatsugatakensis*: *F*_3,9_ = 0.874, Bonferroni-corrected *p* = 0.025).

**Figures 12–19.**
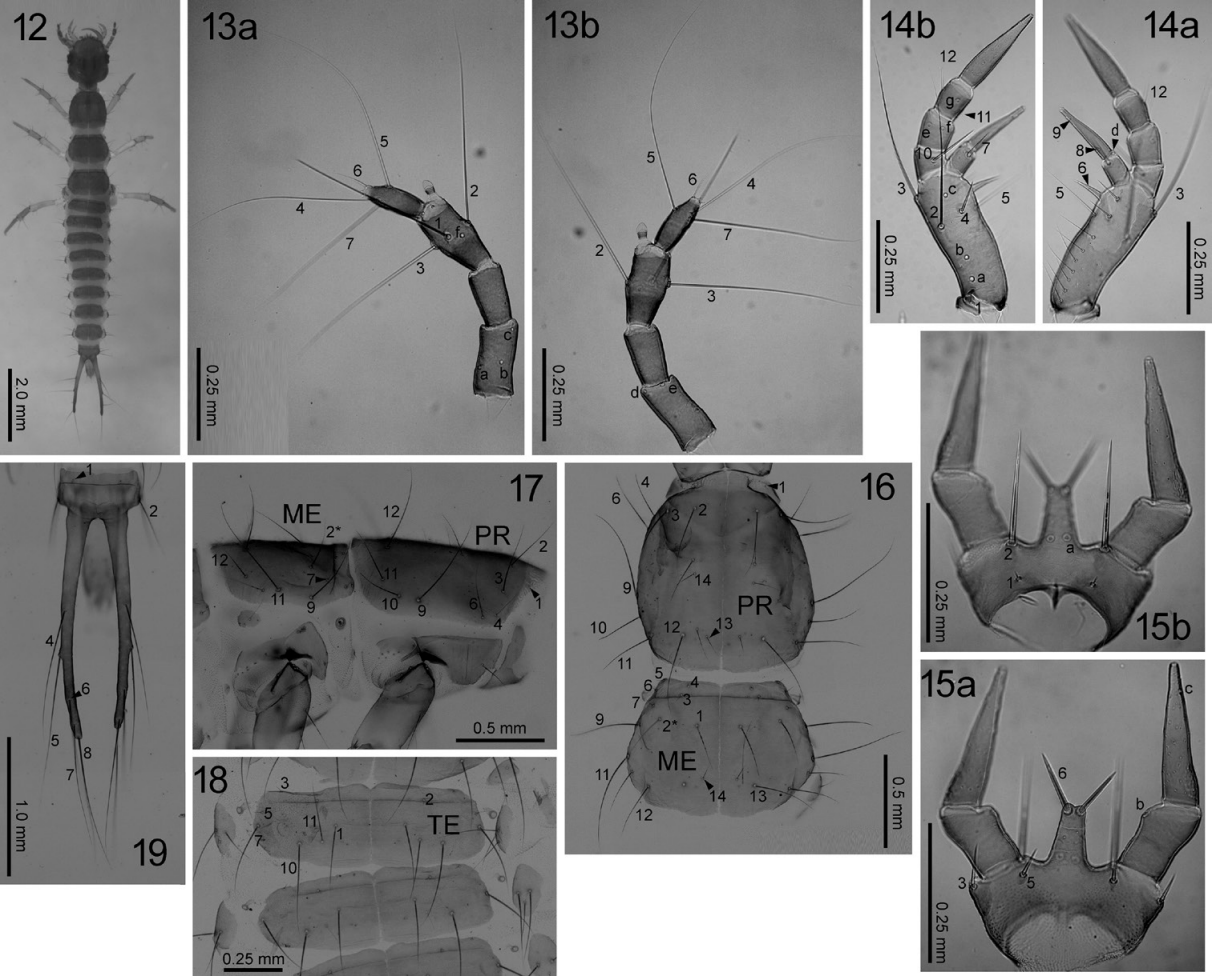
First-instar *Nebria yatsugatakensis* sp. n. **12** Dorsal view **13** antenna (**a** dorsal view; **b** ventral view) **14** maxilla (**a** dorsal view **b** ventral view) **15** labium (**a** dorsal view **b** ventral view) **16** prothorax and mesonotum, dorsal view **17** *Ditto*, right lateral view **18** tergites, dorsal view **19** urogomphi, dorsal view. ME, mesonotum; PR, prothorax; TE, first abdominal tergite. The homology of characters marked with an asterisk (*) is uncertain.

**Figures 20–21.**
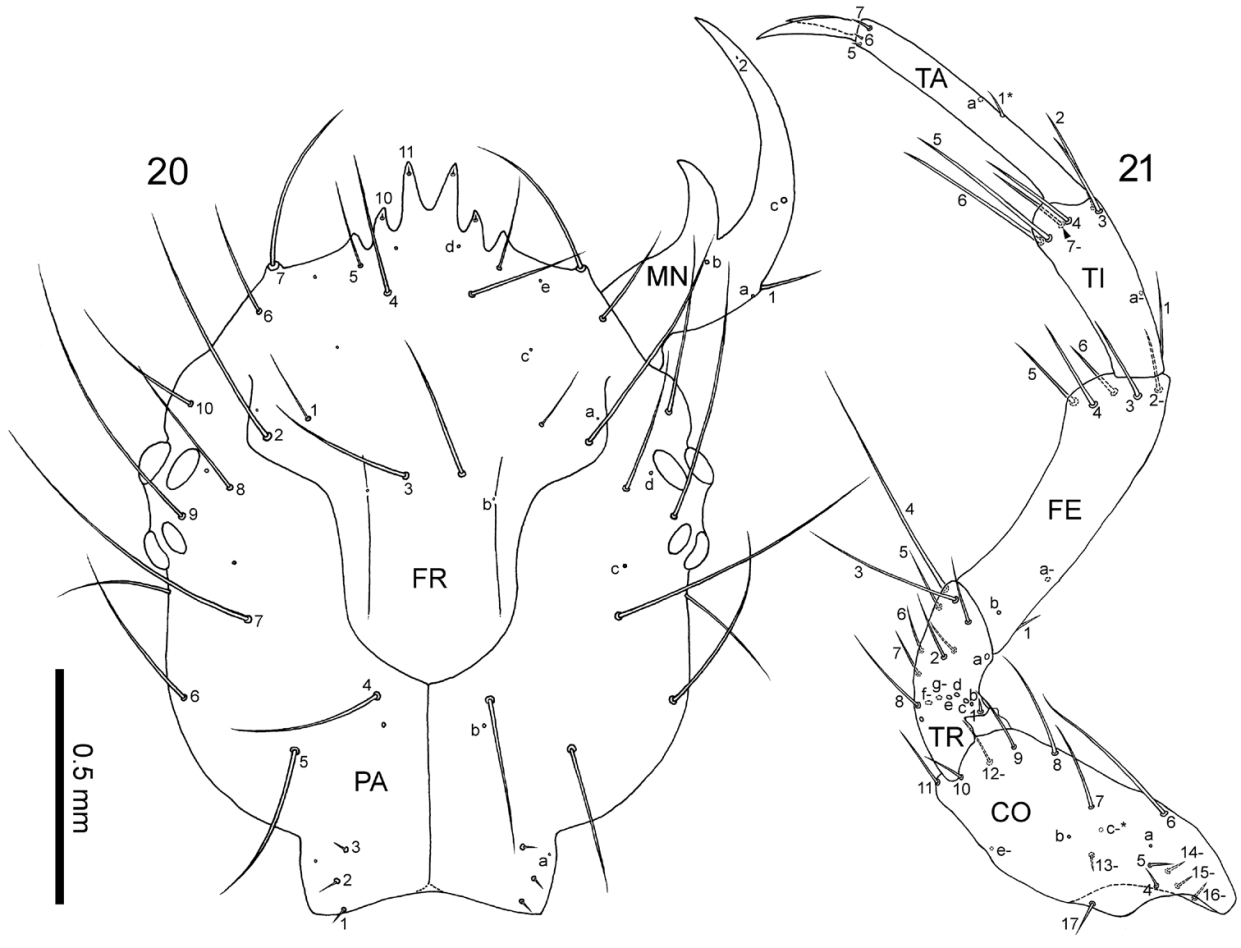
First-instar *Nebria yatsugatakensis* sp. n. **20** Head capsule, dorsal view **21** right foreleg, anterior view. CO, coxa; FE, femur; FR, frontale; MN, mandible; PA, parietale; TA, tarsus; TI, tibia; TR, trochanter. For the foreleg, the homology of characters marked with an asterisk (*) is uncertain, and characters with a hyphen (–) are present on the posterior side.

**Figures 22–28.**
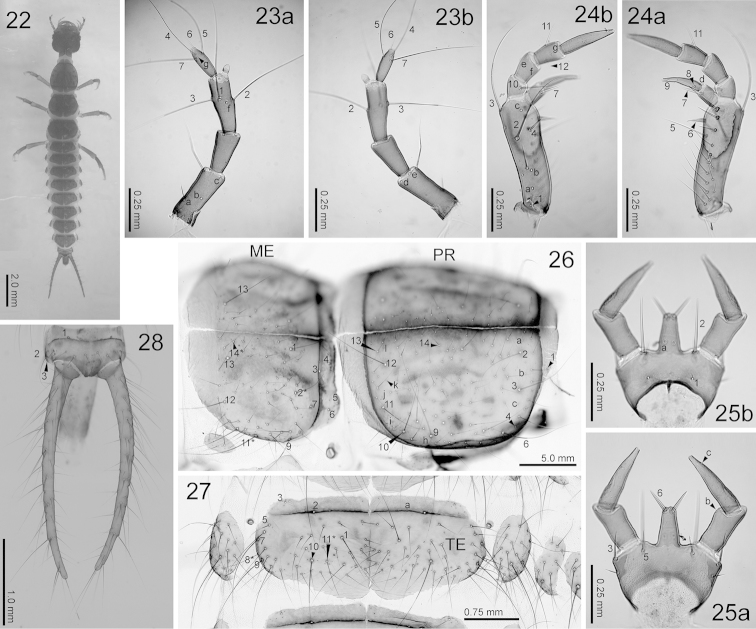
Third-instar *Nebria yatsugatakensis* sp. n. **22** Dorsal view **23** antenna (**a** dorsal view **b** ventral view) **24** maxilla (**a** dorsal view **b** ventral view) **25** labium (**a** dorsal view **b** ventral view) **26** prothorax and mesonotum, right lateral view **27** tergite, dorsal view **28** urogomphi, dorsal view. ME, mesonotum; PR, prothorax; TE, first abdominal tergite. The homology of characters marked with an asterisk (*) is uncertain.

**Figures 29–30.**
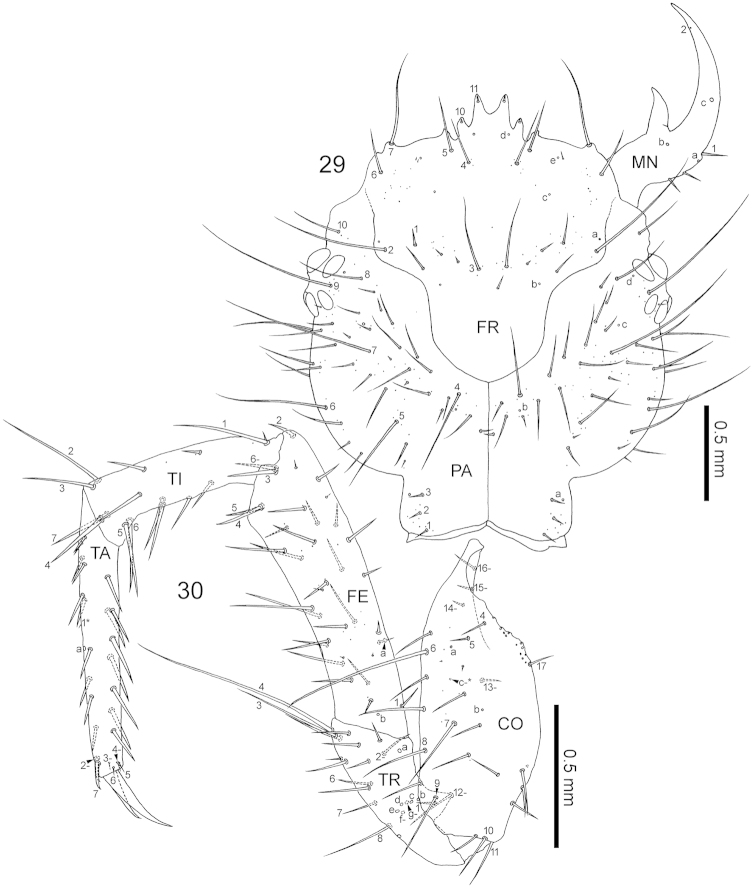
Third-instar *Nebria yatsugatakensis* sp. n**. 29** Head capsule, dorsal view **30** right foreleg, anterior view. CO, coxa; FE, femur; FR, frontale; MN, mandible; PA, parietale; TA, tarsus; TI, tibia; TR, trochanter. For the foreleg, the homology of characters marked with an asterisk (*) is uncertain, and characters with a hyphen (-) are present on the posterior side.

**Figure 31.**
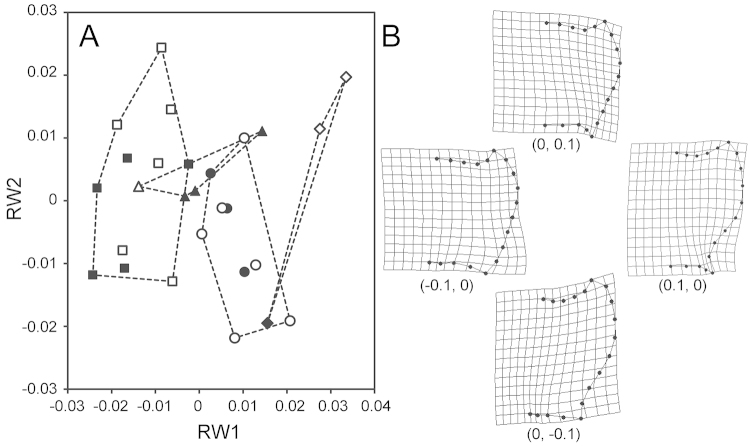
Scatter plot of RW1 and RW2 for the pronotum (**A**) and representations of extreme shape along each axis (**B**). Coordinates of each shape on the plot are presented in parentheses. Triangle- (△), circle- (○), square- (□), and diamond- (◇) marks denote *Nebria quinquelobata* sp. n., *Nebria yatsugatakensis* sp. n., *Nebria chichibuensis*, and *Nebria saeviens*, respectively. Black and white marks denote male and female, respectively. Broken lines represent connections along the margin of each species.

**Figure 32.**
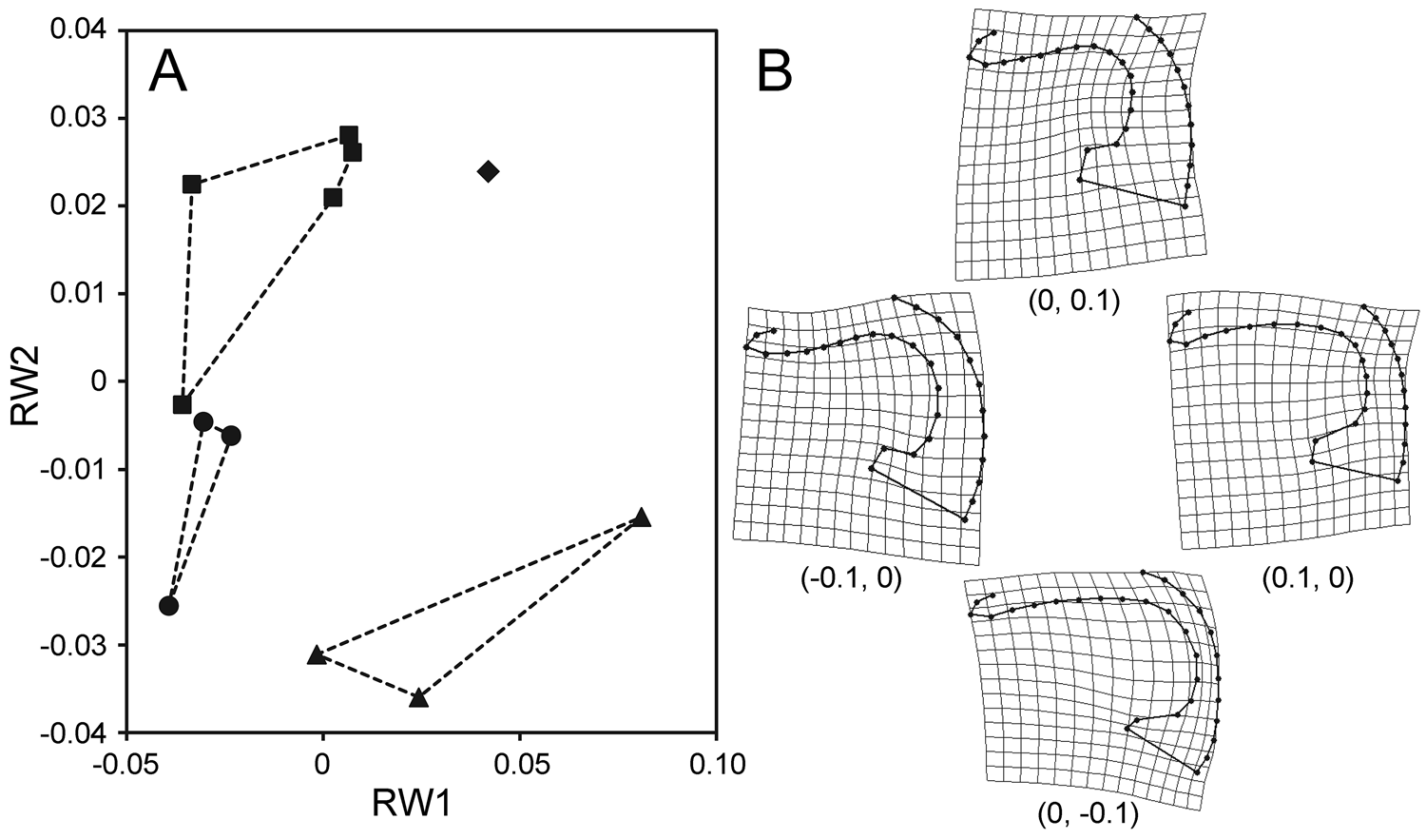
Scatter plot of RW1 and RW2 for the aedeagus (**A**) and representations of extreme shape along each axis (**B**). For explanations of the marks in **A** and of the numerals in parentheses in **B**, see the legend for Fig. 31.

In the aedeagus, RW1 accounted for 48.8% of the total variance and was mainly associated with the relative size (length between landmarks 1 and 31) and the sinuation (curvature of landmarks 5–10) of the subapical portion. RW2 accounted for 30.0% of the total variance and was associated mainly with slenderness, particularly of the basal half. On the scatter plot, four species were clearly segregated and did not overlap. Results of Procrustes ANOVA revealed that the overall difference was significant (*F*_3,7_ = 2.586, Bonferroni-corrected *p* < 0.001). Post hoc tests indicated that *Nebria quinquelobata* and *Nebria saeviens* were significantly different (*F*_3,4_ = 1.043, Bonferroni-corrected *p* = 0.045), with *Nebria yatsugatakensis* being intermediate (vs. *Nebria saeviens*: *F*_3,4_ = 0.865, Bonferroni-corrected *p* = 0.101; vs. *Nebria quinquelobata*: *F*_3,2_ = 0.746, Bonferroni-corrected *p* = 0.256).

## Discussion

In *Sadonebria*, nine new species have recently been separated from known species based on differences in the shape of the endophallus (Sasakawa and Kubota 2006; Sasakawa 2008, 2009, 2010; Sasakawa and Toki 2011). These nine species were all previously recognized as *Nebria sadona*. In contrast, one of the new species described here, *Nebria quinquelobata*, had been recognized as *Nebria saeviens*. The occurrence of this type of cryptic species (i.e., species that are clearly distinguishable only by the shape of the endophallus) in that other than *Nebria* “*sadona*” may indicate that other such cryptic species remain to be discovered in the subgenus. Future studies are needed to revise other consubgeneric species (e.g., *Nebria chinensis* Bates, 1872 and *Nebria niitakana* Kano, 1930), as well as other populations recognized as *Nebria saeviens* (Yoshitake et al. 2011) based on the same morphological traits (endophallus of male genitalia). It is also important to assess the utility of this genital morphology for species-level taxonomy in other *Nebria* subgenera.

The results of geometric morphometrics complement those of the comparative morphology of the male endophallus. For example, morphometric values for both the pronotum and aedeagus were clearly different between *Nebria quinquelobata* and *Nebria saeviens*, and these differences were statistically supported. Although the difference in the pronotum between *Nebria yatsugatakensis* and *Nebria chichibuensis* was not statistically supported, the two species were largely segregated on the scatter plot and only partially overlapped. In the analysis of the aedeagus, the two species are clearly segregated, and this difference was comparable to that between *Nebria quinquelobata* and *Nebria saeviens*, for which the morphometric difference was statistically supported. Some of the statistical insignificances in the results of geometric morphometrics would be attributed to the small sample size of some species. Future studies need to address this issue by re-analysis using additional materials. Importantly, the geometric morphometrics performed here are based on external and genital structures that had been virtually neglected in recent studies of *Sadonebria*. Nevertheless, these results discriminate among very similar species, with an accuracy close to that of comparative morphology of the male endophallus. Thus, geometric morphometrics can provide insights into future studies of *Sadonebria* taxonomy. For example, geographic variation in widely-distributed species can be examined by this morphometric technique.

In the genus *Nebria*, larvae of species from the following 10 subgenera have been described [subgeneric taxonomy follows Ledoux and Roux (2005)]: *Alpaeonebria* Csiki, 1946; *Paranebria* Jeannel, 1937; *Boreonebria* Jeannel, 1937; *Nebria* Latreille, 1802; *Oreonebria* Daniel, 1903; *Paranebria* Jeannel, 1937; *Nebriola* Daniel, 1903; *Eunebria* Jeannel, 1937; *Tyrrhenia* Ledoux & Roux, 2005; and *Nippononebria* Uéno, 1955 (Emden 1942; Habu 1958; Kurosa 1959; Andersen 1970; Luff 1972, 1993; Spence et al. 1976; Arndt 1991; Šustek 1993; Huber and Molenda 2004). Compared with these species, the larvae of *Nebria yatsugatakensis* are unusual in that the thoracic nota and abdominal tergites of older instars have numerous secondary setae on the entire surface. In species of the other subgenera, thoracic nota and abdominal tergites of older instars have no or few secondary setae, described by Luff (1993) as a generic character of the genus *Nebria* (but this character was not described in species of *Alpaeonebria*, *Orientonebria*, *Paranebria*, *Tyrrhenia*, or *Nippononebria*). This unusual condition found in larval thoracic nota and abdominal tergites of *Nebria yatsugatakensis* could be an autapomorphy of *Sadonebria*, because the same character state was found in a field-collected larva that is probably another *Sadonebria* species, *Nebria shikokuensis* Sasakawa, 2011, based on morphological similarities and collection site (Sasakawa, unpublished data). To test this assumption, larvae of additional species of *Sadonebria* and the subgenus *Eonebria* Semenov & Znojko, 1928, the putative sister taxon of *Sadonebria* (Ledoux and Roux 2005), need to be examined. The results could provide insights into the subgeneric taxonomy of *Sadonebria*, as well as the taxonomic importance of larval secondary setae, which has not been addressed in the subgeneric taxonomy of the genus *Nebria*.

## Supplementary Material

XML Treatment for Nebria (Sadonebria) quinquelobata

XML Treatment for Nebria (Sadonebria) yatsugatakensis
